# Distribution and genetic diversity of *Southern rice black-streaked dwarf viru*s in China

**DOI:** 10.1186/1743-422X-10-307

**Published:** 2013-10-16

**Authors:** Zhaobang Cheng, Shuo Li, Ruizhen Gao, Feng Sun, Wancai Liu, Guohui Zhou, Jianxiang Wu, Xueping Zhou, Yijun Zhou

**Affiliations:** 1 Institute of Plant Protection, Jiangsu Academy of Agricultural Sciences, Jiangsu Technical Service Center of Diagnosis and Detection for Plant Virus Diseases, Nanjing 210014, P. R. China; 2 National Agro-Tech Extension and Service Center, Beijing 100125, P. R. China; 3 College of Natural Resources and Environment, South China Agricultural University, Guangzhou 510642, P. R. China; 4 State Key Laboratory of Rice Biology, Institute of Biotechnology, Zhejiang University, Hangzhou 310058, P. R. China

**Keywords:** Southern rice black-streaked dwarf virus, Viral distribution in China, Genetic diversity of SRBSDV

## Abstract

**Background:**

Rice and maize dwarf diseases caused by the newly introduced *Southern rice black-streaked dwarf virus* (SRBSDV) have led to severe economic losses in South China in recent years. The distribution and diversity of SRBSDV have not been investigated in the main rice and maize growing areas in China. In this study, the distribution of SRBSDV in China was determined by using reverse transcription-polymerase chain reaction (RT-PCR).

**Results:**

Between 2009 and 2010, 2404 plant samples (2294 rice, 110 maize samples, and more than 300 cultivars) with dwarf symptoms were collected from fields in 194 counties of 17 provinces in China and SRBSDV was detected. The results indicated that 1545 (64.27%) of samples (both rice and maize) were infected with SRBSDV. SRBSDV was detected widely in Hainan, Guangdong, Guangxi, Yunnan, Guizhou, Chongqing, Fujian, Jiangxi, Hunan, Hubei, Anhui, Jiangsu, and Zhejiang provinces, which suggests SRBSDV is an important pathogen causing rice dwarfing diseases in South China. Phylogenetic analysis of 15 representative virus isolates revealed that SRBSDV isolates in China had high levels of nucleotide and amino acid sequence identities (>97.8%).

**Conclusions:**

SRBSDV spreads naturally in Yangtze River basin and south region, the location of the major rice production areas. In comparison, the virus rarely spreads north of Yangtze River in North China. Distribution of SRBSDV is consistent with the migrating and existing ranges of its vector WBPH, suggesting that SRBSDV might be introduced into South China along with the migration of viruliferous WBPH.

## Background

*Southern rice black-streaked dwarf virus* (SRBSDV) was first identified in Yangjiang, Guangdong province in China in 2001, and has been proposed as a new member of the genus *Fijivirus* in the family *Reoviridae*[1]. Infected rice plants show typical dwarf symptoms, along with dark greening of the leaves, pronounced stunting, twisting of leaf tips, and small white waxy galls along veinlets on the underside of leaf blades and culms [1,2]. SRBSDV can infect rice causing distinct symptoms at different growth stages, and it can also infect maize (*Zea mays*), *Coix chinensis*, *Echinochloa*, *Juncellus* and *Pennisetum*[3]. SRBSDV is transmitted mainly by the white back planthopper (WBPH, *Sogatella furcifera* Horváth) in a persistent, circulative-propagative manner. The virus, however, cannot be transmitted from WBPH female adults to their progeny via eggs [1]. The small brown planthopper (SBPH, *Laodelphax striatellus* Fallén), a major vector of the *Rice black-streaked dwarf virus* (RBSDV) [4], which is most closely related to SRBSDV in phylogeny, can acquire SRBSDV but not transmit it [5]. Available data also shows that WBPH is the major vector of SRBSDV with high efficiency of transmission in rice fields [1]. Moreover, the virus is not transmitted through seed [6].

SRBSDV shares major similarities of symptoms, host ranges, virion morphology, and serology with RBSDV [1,2], another member of the genus *Fijivirus*. RBSDV was widespread and prevalent in most parts of East, North, Northwest, and Northeast China in the last century [4,7]. The two viruses are indistinguishable in agarose or polyacrylamide gel electrophoretic profiles of their genome segments [1]. Due to their overlapping plant host ranges and nearly indistinguishable symptoms in their common plant hosts, it is not easy to distinguish these two virus diseases based on visual symptoms. Recently, the complete nucleotide sequences of SRBSDV and RBSDV genomic RNAs were determined. Ten segments of SRBSDV share 60-80% of nucleotide sequence identities with RBSDV’s counterparts [8-10], facilitating accurate identification of the two viruses by using molecular approaches.

SRBSDV causes a devastating disease that threatens rice production in many provinces in China. In 2009, the rice black-streaked dwarf disease caused by SRBSDV resulted in significant losses in late rice production in South China, especially in Hunan, Hubei, Guangxi, and Jiangxi provinces [11,12]. In 2010, SRBSDV broke out again in these regions. At the same time, SRBSDV also caused severe losses in northern Vietnam, the overwinter location of WBPH [13,14]. Accurate identification of the virus and its distribution is the first step in designing effective disease control strategies. To date, however, the available information about the occurrence of SRBSDV in China is limited, given that related investigations were only performed within confined geographic areas. In this paper, we collected 2404 rice and maize samples from distinct geographic areas of China between 2009 and 2010, and the occurrence of SRBSDV was detected by using RT-PCR.

## Results

### Identification and distribution of SRBSDV in China

**Figure 1:**
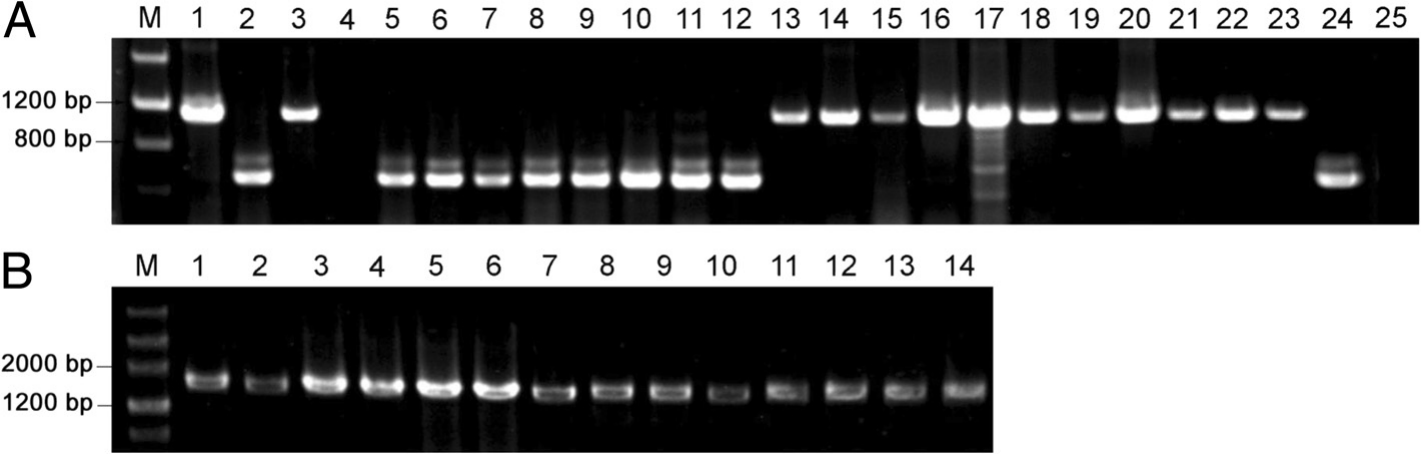
**RT-PCR identification of SRBSDV and RBSDV isolates from rice and maize samples in China. A:** Simultaneous detection of SRBSDV and RBSDV from infected plant samples. Line M, DNA Markers; line 1, RBSDV positive control; line 2, SRBSDV positive control; line 3–24, rice or maize samples from fields; line 25, negative control. **B:** RT-PCR analysis of SRBSDV outer CP genes. Line M, DNA Markers; Line 1–14, partial SRBSDV isolates.

A novel rice black-streaked dwarf disease caused by SRBSDV, exhibiting similar symptoms with another rice dwarfing disease caused by RBSDV in the field, was investigated and identified in China. A duplex RT-PCR was performed to distinguish accurately the two viruses. The 569-bp fragments were produced from SRBSDV-infected plant samples, whereas the 1119-bp products were amplified from samples infected by RBSDV. No amplified products were detected from healthy control plants (Figure 1A). Between 2009 and 2010, 2404 rice and maize samples with typical dwarf symptoms were collected and identified from 194 counties in 17 provinces in China. The results showed that SRBSDV was detected in 1537 out of 2294 rice and in 8 out of 110 maize samples, and positive samples were from 166 counties of 14 provinces, excluding Shanghai, Shandong and Hebei (Table 1). The total detection rate of SRBSDV was 64.27%. Precisely, the detection rate was 67.00% in rice samples, and 7.27% in maize plants.

**Table 1:** RT-PCR detection of SRBSDV and RBSDV in samples collected from different geographic regions in China

**Sample origin**^**a**^		**Total samples**	**SRBSDV**					**RBSDV**					**Co-infection**
**Provinces**	**Total counties**		**Rice**	**Positive**	**Maize**	**Positive**	**Detection rate (%)**	**Rice**	**Positive**	**Maize**	**Positive**	**Detection rate (%)**	
Shandong	7	87	18	0	69	0	0.00	18	17	69	46	72.41	0
Anhui	23	273	256	119	17	0	43.59	256	85	17	0	31.14	0
Hunan	15	141	141	126	0	0	89.36	141	0	0	0	0.00	0
Jiangxi	17	192	192	134	0	0	69.79	192	23	0	0	11.98	0
Jiangsu	22	235	231	5	4	0	2.13	231	130	4	4	57.02	0
Guangxi	29	493	490	412	3	0	83.57	490	0	3	0	0.00	0
Guangdong	22	439	439	372	0	0	84.74	439	0	0	0	0.00	0
Zhejiang	7	38	32	26	6	2	73.68	32	5	6	0	13.16	0
Fujian	9	140	134	119	6	6	89.29	134	0	6	0	0.00	0
Hubei	13	65	65	56	0	0	86.15	65	0	0	0	0.00	0
Sichuan	1	11	11	1	0	0	9.09	11	0	0	0	0.00	0
Yunnan	10	48	48	31	0	0	64.58	48	0	0	0	0.00	0
Shanghai	5	12	12	0	0	0	0.00	12	10	0	0	83.33	0
Chongqing	3	18	18	13	0	0	72.22	18	0	0	0	0.00	0
Guizhou	6	74	74	69	0	0	93.24	74	0	0	0	0.00	0
Hainan	3	133	133	54	0	0	40.60	133	0	0	0	0.00	0
Hebei	2	5	0	0	5	0	0.00	0	0	5	5	100.00	0
Total	194	2404	2294	1537	110	8	64.27	2294	270	110	55	13.52	0

^a^Total 2404 samples were collected from different geographic areas in China between 2009 and 2010.

**Figure 2:**
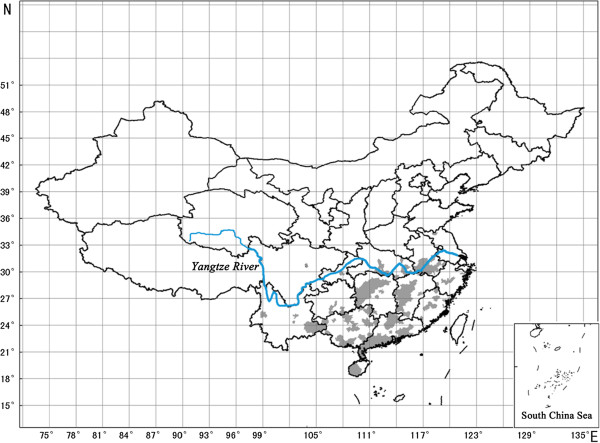
**Distribution of SRBSDV in China between 2009 and 2010.** Locations (counties) where SRBSDV was found are marked as gray shadows.

On the basis of results from molecular diagnosis, the distribution map of SRBSDV in China was plotted (Figure 2). As is shown in Figure 2, the virus disease occurred throughout most parts of Hainan, Guangdong, Guangxi, Fujian, Jiangxi, Hunan, Hubei, and Anhui provinces, and scattered in southeast parts of Yunnan and Guizhou provinces and southwest parts of Zhejiang province. SRBSDV was only found in a few fields in Sichuan, Chongqing, and Jiangsu provinces. In general, SRBSDV distributed throughout South China, especially in the Yangtze River basin and south of the basin, where hybrid rice is primarily cultivated. In comparison, SRBSDV occurred rarely in northern parts of Yangtze River. However, it was remarkable that five virus isolates were detected from a rice field in north (Xinyi) of Jiangsu province, where japonica rice is widely cultivated, and RBSDV is ubiquitous. Our results reveal that SRBSDV can infect not only hybrid rice, but also conventional rice, and the virus can infect almost all rice varieties in South China. All three main rice types in China, japonica rice, glutinous rice and indica rice, can be infected by SRBSDV. However, the vast majority of cultivars with positive test results were indica rice and hybrid rice in the experiment.

Moreover, detection rate of RBSDV in samples was also counted, and it was found that 11.77% of the rice and 50.00% of the maize samples were infected by RBSDV (Table 1). It was remarkable that plant samples co-infected by SRBSDV and RBSDV were not found. The results indicate that SRBSDV is an important pathogen causing rice dwarf disease in South China, while RBSDV is an important pathogen causing maize rough dwarf disease in maize planting areas of China, such as Shandong and Hebei provinces. Although SRBSDV can also cause maize dwarfing, only a few maize samples were infected by SRBSDV in the experiment.

### Genetic diversity of SRBSDV isolates in China

In order to assess the genetic diversity of SRBSDV isolates in China, segment 10 open reading frames (ORFs, encoding outer Coat Protein [CP]) from partial virus isolates were amplified with the specific primers S10-F/S10-R (Figure 1B). Fifteen representative SRBSDV isolates from rice and maize were selected and sequenced, with at least one sample from each province. The sequence analysis of the outer CP genes revealed that there were high levels of nucleotide and amino acid sequence identities (>97.8%) among the 13 SRBSDV isolates from rice (Table 2). Furthermore, two SRBSDV isolates (ZJDY2 and ZJDY4) from maize (Zhejiang province) also exhibited high identities (>98.4%) with other isolates from rice. In general, all outer CP genes from different host and geographical isolates exhibited high levels of sequence identities. When these sequences were compared with the sequences reported by other laboratories, including four isolates from Vietnam (GU017742, GU017741, GU017740 and GU017739) and five isolates from China (EU523360, HM114212, HM114213, HM114214 and EU784840), more than 97.7% nucleotide identity was observed (data is not shown).

**Table 2:** Nucleotide (top right) and amino acid (bottom left) sequence identities (%) of outer CP genes among SRBSDV isolates from China

	**FJYA4**	**CQFD1**	**AHDZ2**	**ZJWY1**	**ZJDY4**	**ZJDY2**	**YNFN1**	**JXNC135**	**JSXY1**	**HNCX1**	**HNCJ1**	**HBCY6**	**GZSD1**	**GXLC4**	**GDSX1**
FJYA4		98.6	99.1	98.2	98.6	98.7	98.6	98	99.1	99.3	98.4	99.1	98.9	98.9	99.3
CQFD1	98.6		98.9	98	98.4	98.6	98.4	97.8	98.9	99.1	98.2	98.9	98.7	98.7	99.1
AHDZ2	99.1	98.9		98.6	98.9	99.1	98.9	98.4	99.5	99.6	98.7	99.5	99.3	99.3	99.6
ZJWY1	98.2	98	98.6		99.1	99.3	99.1	98.6	98.6	98.7	98.9	98.6	98.4	98.7	98.7
ZJDY4	98.6	98.4	98.9	99.1		99.6	99.5	98.9	98.9	99.1	99.3	98.9	98.7	99.1	99.1
ZJDY2	98.7	98.6	99.1	99.3	99.6		99.6	99.1	99.1	99.3	99.5	99.1	98.9	99.3	99.3
YNFN1	98.6	98.4	98.9	99.1	99.5	99.6		98.9	98.9	99.1	99.3	98.9	98.7	99.1	99.1
JXNC135	98.0	97.8	98.4	98.6	98.9	99.1	98.9		98.4	98.6	98.7	98.4	98.2	98.6	98.6
JSXY1	99.1	98.9	99.5	98.6	98.9	99.1	98.9	98.4		99.6	98.7	99.5	99.3	99.3	99.6
HNCX1	99.3	99.1	99.6	98.7	99.1	99.3	99.1	98.6	99.6		98.9	99.6	99.5	99.5	99.8
HNCJ1	98.4	98.2	98.7	98.9	99.3	99.5	99.3	98.7	98.7	98.9		98.7	98.6	98.9	98.9
HBCY6	99.1	98.9	99.5	98.6	98.9	99.1	98.9	98.4	99.5	99.6	98.7		99.3	99.3	99.6
GZSD1	98.9	98.7	99.3	98.4	98.7	98.9	98.7	98.2	99.3	99.5	98.6	99.3		99.1	99.5
GXLC4	98.9	98.7	99.3	98.7	99.1	99.3	99.1	98.6	99.3	99.5	98.9	99.3	99.1		99.5
GDSX1	99.3	99.1	99.6	98.7	99.1	99.3	99.1	98.6	99.6	99.8	98.9	99.6	99.5	99.5	

*FJYA4*: Yongan, Fujian province; *CQFD1*: Fengdu, Chongqing province; *AHDZ2*: Dongzhi, Anhui province; *ZJWY1*: Wuyi, Zhejiang province. *ZJDY4* and *ZJDY2*: Dongyang, Zhejiang province; *YNFN1*: Funing, Yunnan province; *JXNC135*: Nanchang, Jiangxi province; *JSXY1*: Xinyi, Jiangsu province; *HNCX1*: Chenxi, Hunan province; *HNCJ1*: Changjiang, Hainan province; *HBCY6*: Chongyang, Hubei province; *GZSD1*: Sandu, Guizhou province; *GXLC4*: Luocheng, Guangxi province; *GDSX1*: Suixi, Guangdong province. The number after abbreviation was the numbering of isolate samples. ZJDY4 and ZJDY2 isolates were from maize, and others were from rice.

**Figure 3:**
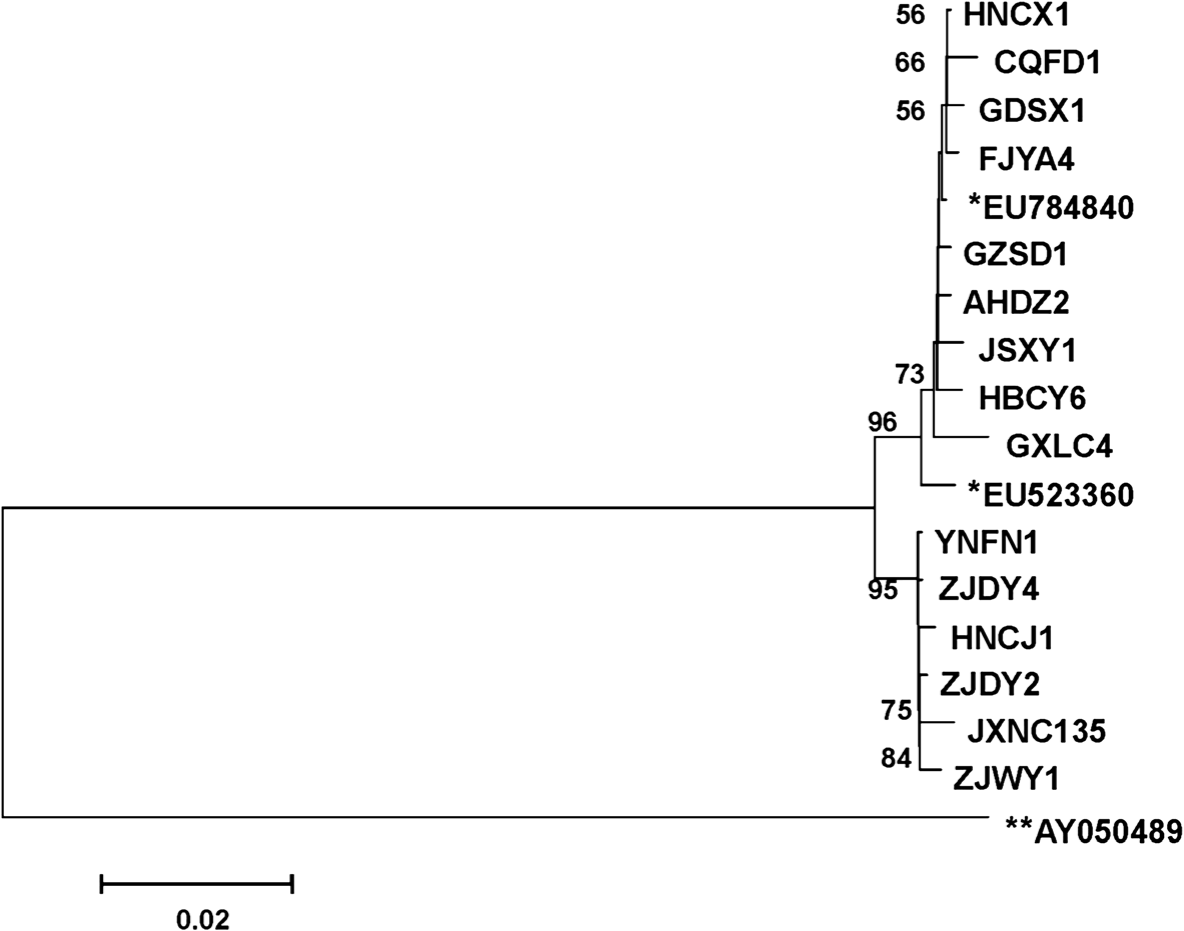
**Phylogenetic analysis of SRBSDV based on outer CP gene nucleotide sequence showing the relationships of SRBSDV isolates from different geographic regions in China.** Trees were constructed by MEGA 4.0 using the neighbor-joining method with 1000 bootstrap replications (bootstrap values were showed where >50%). Trees were rooted with RBSDV segment 10 (AY050489) as outgroup, and two reported SRBSDV isolates (EU784840, EU523360) were also included as references. Bars indicated numbers of nucleotide substitutions per site.

Based on the outer CP gene sequences of SRBSDV isolates detected in the experiment, together with two additional sequences from GenBank (EU784840 and EU523360), the phylogenetic tree was constructed with a RBSDV isolate (AY050489) as the outgroup (Figure 3). No obvious subgrouping could be observed according to hosts or geographical locations of isolates, and all SRBSDV isolates from China were highly similar and clustered in one distinct clade well-separated from the RBSDV isolate. The lower number of nucleotide substitutions per site also showed that there was lower variation among different isolates.

## Discussion

In this paper, the distribution of SRBSDV in China was reported for the first time. Numerous samples from different areas were analyzed for detection of SRBSDV between 2009 and 2010. The results indicate that SRBSDV spreads naturally in the Yangtze River basin and south of the basin, the site of major rice production areas. In comparison, the virus spreads rarely in either north of the Yangtze River or North China, the major sites of maize production, such as Shandong and Hebei provinces.

As a general rule, epidemic and severity of insect-transmission viral diseases on rice depends on the number and viruliferous rate of vector populations, rice varieties, cropping systems, and environment. In 2001, SRBSDV occurred occasionally in Guangdong and Hainan provinces [1]. However, the virus dispersed to neighboring provinces in the next few years and broke out in Hunan and Jiangxi provinces in 2009 [11,12]. The sudden occurrence and development of SRBSDV on rice in South China between 2009 and 2010 could be attributed to the high viruliferous rate of WBPH populations in the advanced-diseased fields. In advanced-diseased fields, the ratio of viruliferous WBPH was as high as 60% [1].

WBPH is a typical long-distance migration pest, and it is widespread in rice fields in China except in the south of Xinjiang province [15,16]. Although a low number of WBPH overwinter in Hainan province and in southern regions of Guangxi, Guangdong and Yunnan provinces in China [17], the main source of spring and summer WBPH in China is in the Indo-China Peninsula, primarily in Vietnam, where the Red River Delta is a direct source and the Mekong Delta is an initial source of WBPH [18]. Every year in China, WBPH from Vietnam migrate regularly to the south regions of 29˚ N along with the southwest air flow and land mainly on the south areas of 25˚ N, where they develop and form dominant populations in the local rice fields [15,17,18]. Our results show that SRBSDV distributes extensively in the south areas of 29˚ N, which is consistent with the primary landing and existing ranges of WBPH. The previously described WBPH migration pattern suggests that SRBSDV might be introduced into South China along with the migration of the viruliferous WBPH.

Furthermore, SBPH can acquire SRBSDV, but not transmit it [5], which indicates that SBPH cannot play a role in SRBSDV epidemics and dissemination. This is a major cause for rare distribution of SRBSDV in the north of the Yangtze River and North China, where RBSDV transmitted by SBPH has a widespread prevalence. SBPH cannot transmit SRBSDV, while WBPH migrates to the south before winter; therefore, the virus has a very slim chance of existing in the Yangtze River basin and most parts of South China in the winter. Because a few numbers of WBPH can overwinter in Hainan and in the south of Guangxi, Guangdong and Yunnan in China, it was considered that SRBSDV disease might exist in these areas in the winter. This assumption was confirmed after our field trips in the winters of 2011 and 2012. This WBPH winter migration pattern suggests that Hainan and the south of Guangxi, Guangdong and Yunnan are the annual distribution regions of SRBSDV in China and that the rest (Yangtze River basin and most parts of South China) are the seasonal distribution regions of SRBSDV.

The accumulation of viruliferous WBPH in wintering grounds and their migration from their source could explain the fast-spreading of SRBSDV in South China. Our results show that all SRBSDV China isolates have higher identities and lower variation, which indicates that SRBSDV might derive from a single origin. Presently, however, little is known about the origin of SRBSDV and if the virus originates from Vietnam. The SRBSDV disease outbreak in northern Vietnam in 2009 [13,14] and the virus isolates from China share high levels of nucleotide and amino acid sequence identities with Vietnam isolates [14], but we have no direct evidence to prove whether or not SRBSDV originates in northern Vietnam. This question is important and remains to be further elucidated.

In recent years, an increasing number of inter-subspecies (japonica-indica) hybrid combinations were cultivated and grown to increase rice yield in South China. A previous study indicated that these japonica-indica hybrids were more favorable for WBPH than japonica rice, and the phenomenon was termed as “super-susceptibility” [19]. Other studies suggested that WBPH-resistance genes retained in sympatric japonica rice landraces in China, while there was little ovicidal and sucking-inhibited resistance to WBPH in either hybrid rice or indica rice. The massive introduction and wide cultivation of susceptible hybrid rice provides a favorable environment for WBPH and increases the possibility for the vector to transmit virus, which is a potential threat to rice production in South China. SRBSDV was found in early, middle-season, and late rice, and its infectivity on early rice was less severe than on middle-season and late rice [3]. The migration of 2nd or 3rd generation viruliferous WBPH, together with viral sources accumulated on early rice, might increase the incidence of SRBSDV on middle-season and late rice.

Though limited maize plants were infected by SRBSDV in our investigation, SRBSDV has the potential to cause severe losses in maize. If SRBSDV spreads to North China, where maize is planted widely, through the migration of WBPH, the virus might threaten maize production as RBSDV has done [5,7]. Notably an isolate of SRBSDV has been obtained from naturally infected maize plants in Shandong province in northern China in 2011 [20], currently the northernmost occurrence of SRBSDV.

In our investigation, all samples had dwarf symptoms, but detection rates of SRBSDV and RBSDV were 64.27% and 13.52% respectively, without co-infection of the two viruses. The remaining samples might be infected by other viruses, such as *Rice ragged stunt virus* (RRSV), *Rice gall dwarf virus* (RGDV), *Rice dwarf virus* (RDV) and *Rice grassy stunt virus* (RGSV). These viruses are common on rice plants in South China, and can also produce dwarf-like symptoms. Of course, positive samples for SRBSDV might be mixed-infected by these viruses. An expanded surveying for all rice virus categories and distribution is needed in the future, which will be helpful for understanding viral epidemiology and controlling disease.

With increasing global temperature and more frequent warm winters, the attacking period of WBPH might be prolonged, and the vector might advance across Vietnam and across China. In addition to the susceptible varieties and cultivation systems, warmer winters will further aggravate the SRBSDV disease occurrence in rice in South China. Therefore, it is urgent to cultivate resistant rice and maize varieties and develop other integrated control strategies for reducing the possibility of damage by SRBSDV in China.

## Materials and methods

### Plant samples

Field surveys were done between 2009 and 2010 in 194 counties of 17 provinces in China, including Guangdong, Guangxi, Hunan, Hubei, Jiangxi, Hainan, Yunnan, Guizhou, Sichuan, Chongqing, Fujian, Zhejiang, Shanghai, Jiangsu, Anhui, Shandong, and Hebei provinces. 2404 cereal crop samples (2294 rice and the rest maize samples) with typical stunting or dwarf symptoms were collected from fields in the above mentioned provinces (104˚19′ E to 121˚8′ E, and 19˚25′ N to 34˚38′ N), and these samples covered more than 300 rice and maize cultivars. At least three rice or maize plants were sampled in each field. Leaf or stem tissues of samples were either used directly for total RNA extraction or frozen at −70°C for further study.

### RNA extraction and primer design

Total RNAs from healthy and virus-infected control plants as well as field samples were extracted following the standard protocol of TRIzol® reagent (Invitrogen, USA). The concentration and quality of each RNA sample were determined with an Eppendorf Biophotometer plus (Eppendorf, Germany). For the simultaneous detection and distinguishing of SRBSDV and RBSDV, a quick duplex RT-PCR analysis was developed as described by Ji et al. (2011) [21]. Three specific primers (S9-SR-F, S9-RB-F and S9-R) were designed according to the reported segment 9 nucleotide sequences of both viruses (GenBank No. AB011403, AF459812, AY050486, AJ291706, AJ297429, AF536564, AF540976, AY039705, EU523359 and EU784843), which shared 75% identity. Primer S9-R (5’-GGATTACAACAHACACAMCGAAA-3’) was complementary to the nucleotide 1469–1491 of segment 9, which was a conserved region in SRBSDV and RBSDV segment 9. Primer S9-SR-F (5’-TTACAYCAAGCACTTTGCGAGG-3’, corresponding to the nucleotide 923–944 of SRBSDV segment 9), and S9-R were used to amplify a 569-bp fragment, which was specific for SRBSDV. Primer S9-RB-F (5’-GRTAGACAGGCAAAYMTAAGCGT-3’) corresponded to the nucleotide 376–398 of RBSDV segment 9, which could also pair with S9-R to amplify a specific 1119-bp fragment for detection of RBSDV. Moreover, primer S10-F (5’-ATGGCTGACATAAGACTTGACAT-3’), corresponding to the nucleotide 22–44 of SRBSDV segment 10 (GenBank No. EU523360), and S10-R (5’-TCATCTGGTGACTTTATTTAACAC-3’), complementary to the nucleotide 1672–1695 of SRBSDV segment 10 (GenBank No. EU784840), were designed to amplify the virus whole outer Coat Protein (CP) gene (1674-bp), which was used to analyze the genetic diversity among different SRBSDV isolates.

### RT-PCR amplification and sequence analysis

First strand cDNA was synthesized with random 6 hexamer as primer by using 1st strand cDNA Synthesis Kit (Invitrogen, USA) according to the manufacturer’s protocols. Subsequent PCR amplification was performed by using *Pfu* DNA polymerase and designed specific primers. In the duplex RT-PCR, primer S9-SR-F, S9-RB-F and S9-R were mixed into the same reaction system to conduct PCR, and then amplified products were separated using 1.5% agarose gel electrophoresis to distinguish simultaneously SRBSDV and RBSDV. The PCR products from S10-F/S10-R primer were cloned individually using pMD18-T vector system (TaKaRa, Dalian, China) and sequenced using an automated dye terminator sequencing system (model 377; PE Applied Biosystems, USA) according to the manufacturer’s protocol. Sequence data was analyzed with DNAstar software. Phylogenetic analysis and molecular diversity among isolates were estimated by calculating the p-distance values for nucleotide and amino acid comparisons using the MEGA program (version 4.0). To determine the relationships between SRBSDV and RBSDV, a phylogenetic tree was constructed via the neighbor-joining (NJ) algorithm with Kimura 2-parameter model.

## Competing interests

The authors declare that they have no competing interests.

## Authors’ contributions

YZ and WL designed the study. ZC, WL, FS, GZ, JW and YZ collected the samples. RG and ZC performed the RT-PCR tests. ZC, SL and YZ analyzed the data. SL, XZ and YZ wrote and finalized the manuscript. All authors read and approved the final manuscript.
